# Phytochemical Contents and Antioxidant and Antiproliferative Activities of Selected Black and White Sesame Seeds

**DOI:** 10.1155/2016/8495630

**Published:** 2016-08-14

**Authors:** Lin Zhou, Xiaohui Lin, Arshad Mehmood Abbasi, Bisheng Zheng

**Affiliations:** ^1^Guangdong Province Key Laboratory for Biotechnology Drug Candidates, School of Biosciences and Biopharmaceutics, Guangdong Pharmaceutical University, Guangzhou 510006, China; ^2^School of Light Industry and Food Sciences, South China University of Technology, Guangzhou 510641, China; ^3^Department of Environmental Sciences, COMSATS Institute of Information Technology, Abbottabad 22060, Pakistan

## Abstract

Sesame (*Sesamum indicum *L.) seeds are popular nutritional food but with limited knowledge about their antioxidant and antiproliferative activities of various varieties. Phytochemical profiles and antioxidant and antiproliferative activities of six varieties of sesame (*Sesamum indicum *L.) seeds were studied.* Fenheizhi3* (black) cultivar exhibited the maximum contents of total phenolics and lignans and values of total oxygen radical absorbance capacity (ORAC) and antiproliferative activity (EC_50_) against HepG2 cells. Bound ORAC values showed strong associations with bound phenolics contents (*r* = 0.976, *p* < 0.01); in bound phenolic extracts, EC_50_ values showed strong negative associations with phenolic contents (*r* = −0.869, *p* < 0.05) and ORAC values (*r* = −0.918, *p* < 0.01). Moreover, the contents of free phenolics were higher than that of the bound phenolics, and the three black sesame seeds generally depicted higher total phenolics compared to the three white varieties. The antioxidant (ORAC values) and antiproliferation activities of six sesame seeds were both associated with contents of bound phenolics (*r* > 0.8, *p* < 0.05). Interestingly, nonlignan components in bound phenolics contributed to the antioxidant and antiproliferative activities. This study suggested that* Fenheizhi3 *variety is superior to the other five varieties as antioxidant supplements.

## 1. Introduction

Sesame “*Sesamum indicum *L.” is a commonly growing plant species mainly in tropical and subtropical regions of the world, particularly in Burma, India, China, and Sudan [1]. Sesame seeds are preferably used along with bread, biscuits, crackers, and so forth and as seasoning in food around the world [2]. Sesame seeds are important sources of oil, protein, carbohydrates, and minerals for human nutrition [3].

Sesame seeds color varied from cream-white to charcoal-black, whereas white and black are the typical skin color. Black sesame seed is superior to white one as food for health in traditional beliefs of Asian countries and is included in* Pharmacopoeia of the People's Republic of China* (PPRC, 2015) as liver and kidney benefiting traditional Chinese medicine (TCM). It has been reported that the seed colors of sesame affect the phytochemical contents and their biological activities [4, 5]. Phytochemical compounds in sesame seed such as sesamin, sesamol, and anthrasesamone F have been proved to have* in vitro/in vivo *antioxidant and antiaging activity [6–9]. Moreover, sesamin and sesamolin showed anti-inflammatory, antihypertensive, and anticarcinogenic effects in numerous studies [10–12].

The common nutritional evaluations of sesame seeds are based on the contents of proteins, oils, and lignans. However, little work was conducted on the correlations between multicomponents and antioxidant activity and multicomponents and antiproliferative activity in various sesame seeds varieties. Therefore, the phytochemical profiles including the contents of phenolics, flavonoids, and lignans,* in vitro* antioxidant activities, and antiproliferative activities against HepG2 cells of six varieties of sesame seeds in China were studied in the present work. Particularly, the six sesame seeds (three of black and three of white skin color, nonscaled planting) are newly bred varieties in midwest of China for improved nutrition values. The knowledge of the antioxidant and antiproliferative activities of various sesame seeds will benefit the sesame seed planters, relevant manufacturers, and ordinary consumers.

## 2. Materials and Methods

### 2.1. Chemicals and Materials

Methanol (MeOH), ethanol (EtOH), n-hexane, ethyl acetate, hydrochloric acid (HCl), and acetic acid (HAC) were purchased from Guanghua Sci-Tech Co., Ltd. (Guangdong, China). Potassium chloride (KCl), sodium acetate (NaAC), sodium carbonate (NaCO_3_), sodium hydroxide (NaOH), potassium phosphate monobasic (KH_2_PO_4_), and potassium phosphate dibasic (K_2_HPO_4_) were of analytical grade and were purchased from Sangon Biotech Co., Ltd. (Shanghai, China). 2,2′-Azobis(2-amidinopropane) dihydrochloride (ABAP), 2′,7′-dichlorofluorescin diacetate (DCFH-DA), fluorescein disodium salt, catechin hydrate (HPLC, ≥98%), Folin-Ciocalteu reagent, ascorbic acid, 6-hydroxy-2,5,7,8-tetramethylchroman-2-carboxylic acid (Trolox), formic acid (chromatographic grade), and methanol (chromatographic grade) were purchased from Sigma-Aldrich, Inc. (St. Louis, MO, USA). Trifluoroacetic acid (TFA, analytical grade) and aluminum chloride (AlCl_3_·6H_2_O, analytical grade) were purchased from Fisher Scientific (Fair Lawn, NJ, USA). Sodium borohydride (NaBH_4_, reagent grade), chloranil (analytical grade), vanillin (analytical grade, 99%), and gallic acid (analytical grade, 99%) were purchased from Aladdin, Inc. (Shanghai, China). Sesamol, sesamolin, and sesamin (HPLC, ≥98%) were purchased from Chengdu Pufei De Biotech Co., Ltd. (Sichuan, China). HepG2 human liver cancer cells were purchased from American Type Culture Collection (Rockville, MD, USA). WME medium, foetal bovine serum (FBS), insulin, and other cell culture reagents were purchased from Gibco Life Technologies Co. (Grand Island, NY, USA). Six sesame seed varieties (three black and three white) were kindly donated by Shanxi Academy of Agricultural Sciences (Taiyuan city, China), which were harvested in 2014 and stored in desiccator at room temperature until analysis.

### 2.2. Moisture Content

The moisture content was analyzed by oven-dry method [13]. Briefly, 1 g of sample was dried in electric oven at 105°C to a constant weight and moisture content was expressed as percent of dry weight (DW) in triplicate.

### 2.3. Phenolics Extraction and Determination

#### 2.3.1. Free Phenolics Extraction

Free phenolic compounds were extracted using the modified method reported previously [14]. Briefly, 1 g of sesame seed was blended with 5 mL of hexane for 1 min. After centrifugation at 2700 ×g for 3 min, residue was defatted two more times. Add 10 mL of 80% chilled acetone to the residue and blend 1 min. The supernatant was collected after being centrifuged at 2700 ×g for 3 min, repeated twice. The supernatant was pooled and evaporated to dryness at 45°C under vacuum. The solution was reconstituted in 10 mL of 70% methanol and free phenolic extracts (100 mg mL^−1^) were stored at −40°C until analysis.

#### 2.3.2. Bound Phenolics Extraction

Bound phenolics were extracted using the method described by Chen et al. [14] with modifications. Briefly, the residues obtained after extraction of free phenolics were flushed with nitrogen gas for 2 min, sealed, and digested with 20 mL of 4 M NaOH at room temperature for 1 h. The mixture was neutralized with 10 M concentrated HCl. Then 20 mL hexane was added to extract residual lipids in the mixture for 10 min (not necessary if no residual lipids). After centrifuging at 2700 ×g for 5 min, remaining residues were extracted five times with ethyl acetate. The ethyl acetate fractions were pooled and evaporated to dryness at 45°C under vacuum. The bound phenolics were reconstituted to 10 mL of 70% methanol and bound phenolic extracts (100 mg mL^−1^) were stored at −40°C until analysis.

#### 2.3.3. Determination of Total Phenolic Contents

Total phenolic contents of each sample were determined using the modified Folin-Ciocalteu colorimetric method [14, 15]. Briefly, free phenolic and bound phenolic extracts were diluted 10–20 times with distilled water and were reacted with Folin-Ciocalteu reagent and then neutralized with Na_2_CO_3_. After 90 min incubation, the absorbance of the resulting solution was recorded at 760 nm using MRX II Dynex plate reader (Dynex Technologies Inc., Chantilly, VA, USA). Total phenolic contents were expressed as grams of gallic acid equivalents per kg of sample on DW (g GAE kg^−1^).

### 2.4. Determination of Total Flavonoid Contents

Total flavonoid contents were determined by sodium borohydride-chloranil (SBC) protocol [16]. Briefly, 1 mL of free and 1 mL of bound phenolic extracts were added to test tubes (15 × 150 mm) and were dried under nitrogen gas. The residues and catechin hydrate standards (0.1–10.0 mM) were prepared in 1 mL of THF/EtOH (1 : 1, v/v). Extracts or standard solution was mixed with 0.5 mL of 50 mM NaBH_4_ solution and 0.5 mL of 74.6 mM AlCl_3_ solution. After 30 min shaking in an orbital shaker at room temperature, 0.1 mL of 50.0 mM NaBH_4_ solution was added with continued shaking for 30 min at room temperature. 0.4 mL of cold 0.8 M acetic acid solution was added and the mixtures were kept in the dark for 15 min after thorough mixing. 0.2 mL of 20.0 mM chloranil was added and the mixture was heated at 95°C with shaking for 60 min. Then, the reaction solutions were cooled using tap water and were brought to 1 mL using methanol. Afterward, 0.2 mL of 16% (w/v) vanillin was added and mixed, followed by addition of 0.4 mL of 12 M HCl and the reaction solutions were kept in the dark for 15 min after thorough mixing. At last, the absorbance was recorded at 490 nm using MRX microplate reader with Revelation workstation (Dynex Technologies, Inc.). Total flavonoid contents were expressed as grams of catechin equivalents per kg of sample on DW (g CE kg^−1^).

### 2.5. Estimation of Lignan Contents

Both the free and bound phenolic extracts (100 mg mL^−1^) were used for determination of lignan contents. The chromatographic analysis was performed to estimate lignan contents in sesame seeds as described by Reshma et al. [17] with some modifications. Briefly, three representative lignans (sesamol, sesamin, and sesamolin) were analyzed for the contents using Waters HPLC system (Waters Corp, Milford, MA) with a Waters C_18_ column (5 *μ*m, 250 mm × 4.6 mm). The mobile phase consisted of methanol/water (75 : 25 v/v) with 0.1% formic acid in water at a flow rate of 1 mL min^−1^. The UV detector was set at 290 nm with column temperature of 30°C and injection volume of 10 *μ*L. Peak was identified by calibration standards of sesamol, sesamin, and sesamolin with a retention time of 3.4, 8.3, and 10.2 min, respectively. The concentration range of standards of sesamol, sesamin, and sesamolin was from 2 to 200 *μ*g mL^−1^ with recovery at 99.50 ± 1.00%, 99.47 ± 1.02%, and 99.62 ± 1.13%, respectively. The results were expressed as milligrams per kg on DW (mg kg^−1^).

### 2.6. Determination of Total Antioxidant Activity

#### 2.6.1. Oxygen Radical Absorbance Capacity (ORAC)

ORAC values were determined using a Fluoroskan Ascent fluorescent spectrophotometer (Molecular Devices, Sunnyvale, CA) [18, 19]. Briefly, 20 *μ*L extracts (100 mg mL^−1^) or Trolox was mixed with 200 *μ*L of fluorescein and incubated at 37°C for 20 min, followed by adding 20 *μ*L of freshly prepared 119.4 mM ABAP into black walled 96-well plates. Fluorescence intensity was recorded automatically at 37°C, excitation of 485 nm, and emission of 535 nm for 35 cycles every 5 min. Final values of ORAC were expressed as micromoles of Trolox equivalents (TE) per gram of sesame seeds on DW basis (*μ*mol TE g^−1^ DW).

#### 2.6.2. Peroxyl Radical Scavenging Capacity (PSC)

PSC values were determined by the method described by Adom and Liu [20]. Briefly, 100 *μ*L extracts (100 mg mL^−1^) or standards solutions (freshly prepared ascorbic acid solutions) were added in each well of the 96-well plate. Then, 100 *μ*L of DCFH dye (13.26 *μ*M), hydrolyzing with 1 mM KOH just before being used in the reaction, was also added. After adding 50 *μ*L of peroxyl radicals producer (ABAP, 40 mM), the mixtures were kept at 37°C for 40 min. Fluorescent was dynamically recorded at excitation of 485 nm and emission of 538 nm using a multimode microplate reader (FilterMax F5, Molecular Devices, USA). The results were calculated as micromoles of vitamin C equivalents (VCE) per gram of sesame seeds on DW basis (*μ*mol VCE g^−1^ DW).

### 2.7. Determination of Antiproliferative Activity

The antiproliferative effects of sesame seeds extracts were assessed by modified methylene blue assay [21]. Briefly, HepG2 human liver cancer cell was grown in WME medium supplemented with 5% FBS, 10 mM N-2-hydroxyethylpiperazine-N′-2-ethanesulfonic acid (HEPES), 2 mM L^−1^ glutamine, 5 g mL^−1^ insulin, 0.05 g mL^−1^ hydrocortisone, 50 units mL^−1^ penicillin, 50 g mL^−1^ streptomycin, and 100 g mL^−1^ gentamycin at 37°C and 5% CO_2_ as described by Liu et al. [22]. HepG2 cells were cultured at a density of 2.5 × 10^4^cells/well in a 96-well microplate with 100 *μ*L of growth medium. After 4 h of incubation at 37°C, growth medium was displaced by 100 *μ*L of fresh medium containing 10–150 mg mL^−1^ of sesame seeds extracts. The wells containing fresh medium without extracts were set as the control. After 72 h of incubation, the number of the viable cells was counted by the methylene blue assay. The antiproliferative effects were assessed by the EC_50_ values and expressed as milligrams of sesame seeds extracts per milliliter.

### 2.8. Statistical Analysis

Statistical analysis was performed using SPSS software version 19.0 (LEAD Technologies, Inc., Chicago, IL). Variation of means was analyzed by ANOVA and Duncan's test. Significance of correlations was calculated by the Pearson coefficient. Dose-Effect analysis was performed using CalcuSyn software, version 2.0 (Biosoft, Cambridge, UK). Statistical significance was set at *p* < 0.05. All data were expressed as the mean ± SD for three replications.

## 3. Results and Discussion

### 3.1. Moisture Content

Moisture content and description of six varieties of sesame seeds are given in Table 1. Except B1 variety, the average moisture content of the other five sesame seed varieties ranged from 4.36 to 5.23%, which was consistent with the published data [3, 13]. The possible reason for the lower moisture content in B1 variety was the moisture loss during storage before transportation.

### 3.2. Phytochemical Composition of Selected Sesame Seeds

#### 3.2.1. Contents of Phenolics and Flavonoids

**Figure 1:**
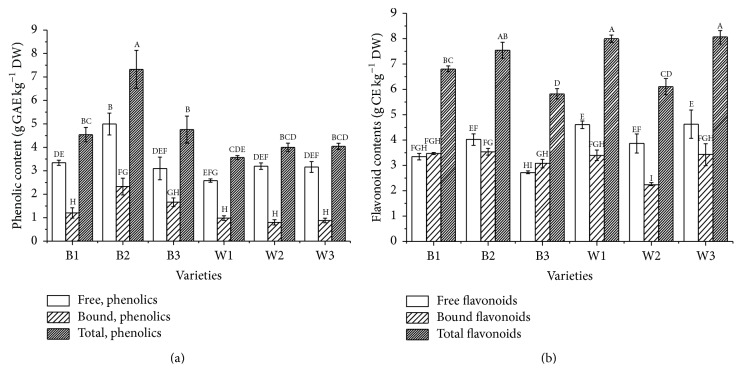
Free, bound, and total phenolic and flavonoid contents in six varieties of sesame seeds (B1, B2, B3, W1, W2, and W3). Bars with no letters in common are significant difference at *p* < 0.05. (a) Phenolic contents. (b) Flavonoid contents.

Measured levels of total phenolic and total flavonoid contents of six sesame seed varieties were presented in Figures 1(a) and 1(b). From Figure 1(a), free phenolic contents were higher than that of the bound phenolic. Black sesame seed variety B2 exhibited maximum free and bound phenolics (4.99 ± 0.47 and 2.33 ± 0.36 g GAE kg^−1^, resp.), while minimum amount was determined in free phenolics of W1 (2.58 ± 0.07 g GAE kg^−1^) and bound phenolics of W2 (0.80 ± 0.11 g GAE kg^−1^). In B2 cultivar, free phenolics were 68.19% and bound phenolics were 31.81% of total phenolic contents. Total phenolic contents in black sesame varieties (B1, B2, and B3) ranged from 4.54 to 7.32 g GAE kg^−1^ with maximum value in B2, whereas among white sesame seed W3 exhibited the highest total phenolic contents of 4.04 ± 0.13 g GAE kg^−1^, followed by W2 (4.00 ± 0.18 g GAE kg^−1^) and W1 (3.56 ± 0.08 g GAE kg^−1^) variety. On the whole, the black sesame varieties contain more phenolics compared to the white varieties. However, no significant differences between free, bound, and total phenolics were observed between black and white sesame varieties. Nadeem et al. [23] reported the total phenolics extracted from sesame cake were 1.72 g GAE kg^−1^. Shahidi et al. [4] reported that the total phenolic contents of two sesame seed cultivars (black and white) were 29.9 ± 0.6 and 10.6 ± 1.6 g catechin equivalents kg^−1^ crude ethanolic extract, respectively. These studies were not comparable with our data for different samples (sesame cake) or reference control (catechin) used in the determination.

From Figure 1(b), the contents of free flavonoid ranged from 2.88 (B3) to 4.61 (W3) g CE kg^−1^, and the bound flavonoids were between 2.24 (W2) and 3.52 (B2) g CE kg^−1^, while total flavonoid contents ranged from 5.80 (B3) to 8.04 (W3) g CE kg^−1^. W3 variety exhibited the highest level of total flavonoids (8.04 ± 0.26) and free flavonoids (4.61 ± 0.06) g CE kg^−1^, while the highest levels of bound flavonoids were present in B2 (3.52 ± 0.08 g CE kg^−1^). On the whole, no significant differences of the flavonoid contents (free, bound, and total flavonoids) were observed between black and white sesame varieties.

#### 3.2.2. Contents of Lignans

**Figure 2:**
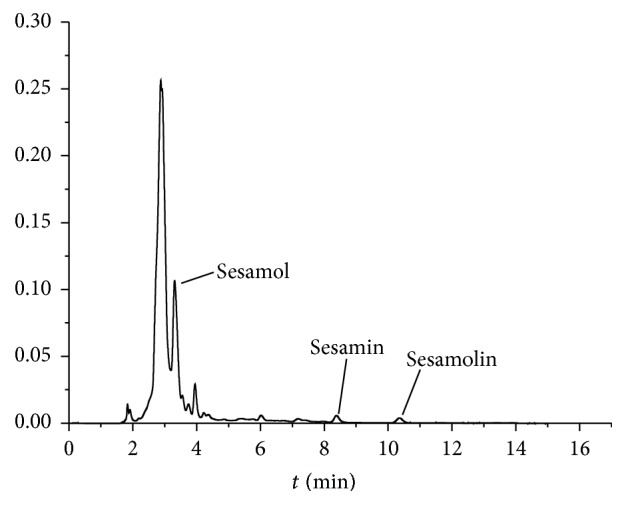
Elution curve of lignans (sesamol, sesamin, and sesamolin) of free phenolic extracts of B2 variety (mobile phase: methanol/water 75 : 25; flow rate: 1 mL min^−1^, wavenumber: 290 nm; and Waters C_18_ column).

Lignans here including sesamol, sesamin, and sesamolin in free and bound extracts of sesame seeds were analyzed by HPLC (Figure 2, Table 2). Overall, free phenolic extracts contain more levels of lignans (>89%) compared to the bound extracts. Sesamol and sesamolin contents were detected in the free extracts of all varieties. The lignans in free phenolic extracts of white and black sesame varieties ranged from 29.28 to 53.76 and 82.83 to 251.91 mg kg^−1^, respectively. The average lignan content of the three black cultivars (167.34 mg kg^−1^) was three times higher than that of the white cultivars (47.54 mg kg^−1^). B2 variety showed the highest sesamol (187.25 ± 10.56 mg kg^−1^) in free phenolic extracts, followed by B3 (121.48 ± 3.28 mg kg^−1^) and B1 (79.21 ± 3.62 mg kg^−1^). In white varieties, sesamol in free phenolic extracts ranged from 7.15 ± 0.75 (W2) to 34.58 ± 1.99 mg kg^−1^ (W1). Likewise, B2 variety had the highest sesamolin content (25.12 ± 0.95* *mg kg^−1^) in free phenolic extracts, followed by B3 (10.30 ± 0.20 mg kg^−1^) and B1 (not detected). Sesamin was detected in the free extracts of four varieties with the highest value in W2 (39.57 ± 1.26 mg kg^−1^) and B2 (39.55 ± 0.38 mg kg^−1^), followed by W1 (14.25 ± 1.74 mg kg^−1^) and B3 (4.95 ± 0.20 mg kg^−1^). However, sesamin was not detected in bound phenolic extracts.

It has been reported [24–26] that sesamin and sesamolin contents in sesame seeds ranged from 1550 to 4200 mg kg^−1^ and 620 to 3590 mg kg^−1^, respectively. In the present study, the possible reason for lower sesamin and sesamolin contents (<40 mg kg^−1^) was the extraction solvents. Although 70~80% methanol is common solvent for the extraction of total phenolics and flavonoids [14, 20], lipid-soluble lignans (sesamin and sesamolin) are partially soluble in 70% methanol based on visual observation. Likewise, the relatively higher content of sesamol compared to sesamin and sesamolin was observed in the free and bound extracts, which is possibly related to the relatively higher dissolving ability of sesamol in 70% methanol. Mostly previous studies were designed to determine the maximal content of lignans in sesame seeds, so the extraction organic solvents commonly used were weak polar ethanol, methanol, and acetonitrile. Here, our aim was to evaluate the lignan levels and their contribution to* in vitro* antioxidant activity in extracted fractions of sesame seeds, which leads to the relatively lower contents of lignans determined. Sesamin and sesamolin were successfully separated in setting chromatographic parameters (Figure 2), but the baseline separation of sesamol was not attained in the present work.

### 3.3. *In Vitro* Antioxidant Activity

Due to the complex components in sesame seeds, two* in vitro* antioxidant activity assays (ORAC and PSC) were used in the present study. The common principle of determination was to measure radical chain breaking ability of antioxidants by monitoring the inhibition of peroxyl radical, while different oxidant-fluorescein probes were used: ABAP-fluorescein in ORAC assay and ABAP-DCFH in PSC assay [18, 20].

**Figure 3:**
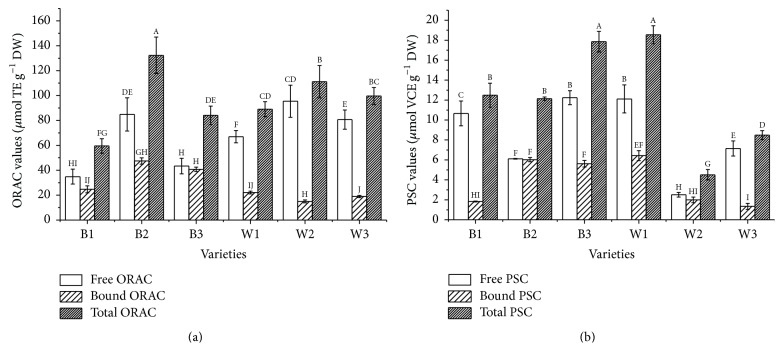
*In vitro* antioxidant activities of six sesame seed varieties (B1, B2, B3, W1, W2, and W3) (mean ± SD; *n* = 3). Bars with no letters in common are significant difference at *p* < 0.05. (a) ORAC values. (b) PSC value.

Measured values of ORAC and PSC based on DW are given in Figures 3(a) and 3(b). ORAC values ranged from 34.85 (B1) to 95.48 (W2) *μ*mol TE g^−1^ in free fractions (Figure 3(a)), while in bound fractions it was between 14.97 (W2) and 40.74 (B3) *μ*mol TE g^−1^. Black sesame variety B2 exhibited the highest total ORAC value (132.33 *μ*mol TE g^−1^), whereas B1 showed the lowest total ORAC value (59.51 *μ*mol TE g^−1^). White sesame variety W1 had the highest total PSC value among all varieties, which was 4 times higher than the lowest one (W2), followed by B3, B1, B2, W3, and W2 (Figure 3(b)). PSC value of free fractions ranged from 2.55 (W2) to 12.27 (B3) *μ*mol VCE g^−1^, while in bound fractions it ranged from 1.33 (W3) to 6.45 (W1) *μ*mol VCE g^−1^. The total PSC values ranged from 4.50 (W2) to 18.55 (W1) *μ*mol VCE g^−1^. Ishiyama et al. [27] reported that ORAC values of two sesame seed varieties (Japan Kanto No. 1 and Gomazou) were 658.3 and 8.27 mg TE g^−1^, respectively. These values correspond to 26.30 and 33.04 *μ*mol TE g^−1^ of sesame seed, which was comparable to the free fractions of B1 and B3 but was nearly one-fourth to one-third of the corresponding values of varieties B2, W1, W2, and W3. Othman et al. [28] recently reported that the average ORAC values of polar-soluble crude extracts for white and gold sesame seed were 347.20 and 217.00 *μ*mol TE g^−1^, respectively. These reported data were nearly 2.6- and 1.6-fold higher than total ORAC value in B2 (the highest in this study), which verified the significant antioxidant activity of polar-soluble extracts.

Correlation analysis of the* in vitro* antioxidant activity with the multicomponents of selected sesame seeds was shown in Table 3. Bound ORAC values showed strong associations with contents of bound phenolics (*r* = 0.976, *p* < 0.01). Moreover, total ORAC values showed strong association with contents of total phenolics (*r* = 0.701, *p* < 0.01). Moderate associations were observed between total ORAC values and contents of total flavonoids (*r* = 0.412, *p* < 0.05). However, associations between total lignans and antioxidant activities (ORAC and PSC values) were not observed. From ORAC values of B2, W1, W2, and W3, PSC values of B1, B3, W1, and W3, and their corresponding contents of lignans (Figure 3, Table 3), it can be inferred that the relatively higher antioxidant activities of free phenolic extracts compared to bound phenolic extracts were related to the relatively higher contents of lignans in free phenolic extracts. Comparable antioxidant activity was observed in PSC values of B2 and W2 variety and ORAC values of B2 and B3 variety in free and bound phenolic extracts (Figure 3, Table 3), even none or less lignans determined in bound phenolic extracts, which indicated that nonlignan components contribute to their corresponding antioxidant activities. There were no significant associations between PSC values (free, bound, and total) with corresponding contents of phenolic and flavonoid. Though specific reason is not clear, synergistic effects of various phytochemicals may involve complex antioxidant behavior of these constituents. Many similar reports were observed in selected components of sesame seed or sesame seed oil, such as synergistic antioxidant activity between lignans (sesamin, sesamol, sesamolin) and tocopherols (*α*, *β*, and *γ*) in various test system [29, 30]. Except the weak polar lignan components, the water soluble extracts of sesame seed raised concern. Moazzami et al. [31] quantified lignan glucosides in 65 different sesame seed cultivars, but no significant difference between black and white seeds was observed. Othman et al. [28] reported that extracts from white sesame seed had relatively higher antioxidant capacity compared to extracts from gold sesame seeds and ascribed the possible reason to the differences in the contents of antioxidants. Ide et al. [32] compared the physiological activities of sesame seeds with different concentration of lignans in rats and found that sesame seeds rich in lignans, irrespective of composition of lignans, greatly affect hepatic fatty acid oxidation and serum triacylglycerol levels. However, the researchers stated that there is a possibility that compounds other than lignans are involved in the physiological activities of tested sesame seeds. These reports indicate the complex antioxidant activity of multicomponents of sesame seeds.

### 3.4. Antiproliferation in HepG2 Cancer Cell

Cell culture models provide efficient approach that imitates the uptake, distribution, and metabolism of antioxidant compounds* in vivo* [33], and human liver cancer HepG2 cell model is widely used in the antioxidant, antiproliferative activity of various compounds [14, 34, 35]. The antiproliferative effect of free and bound phenolic extracts against HepG2 cells was present in Table 4. The EC_50_ of free phenolic extracts ranged from 21.04 ± 0.60 to 124.91 ± 2.79 mg mL^−1^ for HepG2 cells, while corresponding values for bound phenolic extracts ranged from 23.57 ± 0.88 to 109.53 ± 1.23 mg mL^−1^. Lower values of EC_50_ indicate a higher antiproliferative activity. Both free and bound phenolic extracts of B2 variety showed antiproliferative activity. This result is consistent with the relatively higher contents of phenolics, flavonoids, and lignans and ORAC values of B2 variety. Interestingly, the black sesame seed varieties showed higher antiproliferative activity compared to the white varieties in both free phenolic and bound phenolic extracts. Moreover, in bound phenolic extracts, highly negative significant correlations were observed between EC_50_ and bound phenolics (*r* = −0.869, *p* < 0.05) and EC_50_ and bound ORAC values (*r* = −0.918, *p* < 0.01); in free phenolic extracts, highly negative significant correlations were observed between EC_50_ and sesamol contents (*r* = −0.858, *p* < 0.05) (data not shown).

Sesamin and sesamol have been reported to have anticancer activities in many cancer cell lines including HepG2. Sesamin of 25–125 *μ*M could induce HepG2 cell death involving apoptosis [36]. Content of sesamin in free phenolic extract of B2 variety was nearly 39.55 mg kg^−1^ DW, which is roughly equivalent to 11 *μ*M sesamin. As for sesamol, at dosage of 50 *μ*M, apoptosis against HepG2 cell could be observed by Liu et al. [37], while the content of sesamol in free phenolic extracts of B2 variety was nearly 187.25 mg kg^−1^ DW, which is roughly equivalent to 135 *μ*M of sesamol. These results were consistent with the significant correlation observed between antiproliferation activity and content of sesamol in free phenolic extracts. Additionally, in bound phenolic extracts of B1, B2, W1, and W2 variety, contents of lignans (sesamol, sesamin, and sesamolin) were far less compared to their free phenolic extracts (Table 2). However, the corresponding antiproliferative activities in free phenolic extracts and bound phenolic extracts were comparable (Table 4). Thus, it can be inferred that the antiproliferative activities of sesame seed were not only related to the contents of phenolics and lignans (sesamol) of free phenolic extracts but also related to nonlignan components in bound phenolic extracts, which need further investigation.

## 4. Conclusions

Sesame seed variety of* Fenheizhi3* (black, B2) could be more valuable than the others (*Zhenzhou1*,* 05H27*,* Jizhi157*,* Fenzhi2*, and* Jizhi1*) based on the* ex vivo* antioxidant and antiproliferative activities. In selected sesame seeds, free phenolic contents were higher than the bound phenolic contents; black sesame seeds generally depicted higher contents of total phenolic compared to the white varieties. Moreover, the antioxidant activities (total ORAC values) were associated with the contents of total phenolics and flavonoids (*r* > 0.4, *p* < 0.05); the antiproliferation activities (bound EC_50_) were associated with contents of bound phenolics and sesamol (*r* > 0.8, *p* < 0.05). Additionally, nonlignan components in bound phenolic extracts contributed to the corresponding antioxidant and antiproliferative activities.

## Supplementary Material

 The inhibition of HepG2 cells proliferation by the free and bound fractions were observed in a dose-dependent manner in Figure S1. The EC_50_ of free phenolic extracts ranged from 21.04 ± 0.60 to 124.91 ± 2.79 mg mL^−^^1^ for HepG2 cells, while corresponding values for bound phenolic extracts ranged from 23.57 ± 0.88 to 109.53 ± 1.23 mg mL^−^^1^. Lower values of EC_50_ indicate a higher antiproliferative activity. Both free and bound phenolic extracts of B2 variety showed highest antiproliferative activity.

## Figures and Tables

**Table 1:** Description and moisture contents of six sesame seeds varieties.

Varieties	Abbreviation	Harvest year	Color	Origin	Moisture content (%)
*Zhenzhou1*	B1	2014	Black	Zhenzhou, Henan	1.63 ± 0.13
*Fenheizhi3*	B2	2014	Black	Agriculture academy of Shanxi	5.14 ± 0.09
*05H27*	B3	2014	Black	Shanxi Academy of Agricultural Sciences	5.22 ± 0.01
*Jizhi157*	W1	2014	White	Hebei	4.50 ± 0.14
*Fenzhi2*	W2	2014	White	Shanxi	4.63 ± 0.13
*Jizhi1*	W3	2014	White	Shanxi Academy of Agricultural Sciences	4.63 ± 0.03

Data were reported as mean ± SD.

**Table 2:** Lignans (sesamol, sesamin, and sesamolin) contents of free and bound phenolics extracts in six sesame seeds varieties.

Varieties	Sesamol (mg kg^−1^)		Sesamin (mg kg^−1^)		Sesamolin (mg kg^−1^)		Total (mg kg^−1^)
	Free extracts	Bound extracts	Free extracts	Bound extracts	Free extracts	Bound extracts	
B1	79.21 ± 3.62	7.14 ± 0.23	ND	ND	ND	ND	86.35
B2	187.25 ± 10.56	30.54 ± 2.98	39.55 ± 0.38	ND	25.12 ± 0.95	ND	282.46
B3	121.48 ± 3.28	ND	4.95 ± 0.20	ND	10.30 ± 0.20	ND	136.72
W1	34.58 ± 1.99	ND	14.25 ± 1.74	ND	4.94 ± 0.21	ND	53.76
W2	7.15 ± 0.75	ND	39.57 ± 1.26	ND	12.86 ± 0.76	ND	59.57
W3	27.32 ± 3.46	ND	ND	ND	1.97 ± 0.22	ND	29.28

ND means not detected.

Data were reported as mean ± SD.

Limit of detection was 0.06 mg kg^−1^ for sesamol, 0.83 mg kg^−1^ for sesamin, and 1.01 mg kg^−1^ for sesamolin.

**Table 3:** Correlation of multicomponents (phenolics, flavonoids, and lignans) with antioxidant (ORAC, PSC) and antiproliferation (EC_50_) activities of six sesame seed varieties.

	Free phenolics	Bound phenolics	Total phenolics	Free flavonoids	Bound flavonoids	Total flavonoids	Lignans
Free PSC values	−0.406	NC	NC	−0.265	NC	NC	NC
Bound PSC values	NC	0.604	NC	NC	0.294	NC	NC
Total PSC values	NC	NC	0.020	NC	NC	0.130	0.542
Free ORAC values	0.286	NC	NC	0.634	NC	NC	NC
Bound ORAC value	NC	0.976^*∗∗*^	NC	NC	0.417	NC	NC
Total ORAC values	NC	NC	0.701^*∗∗*^	NC	NC	0.412^*∗*^	0.533
Free EC_50_	−0.750	NC	NC	0.465	NC	NC	NC
Bound EC_50_	NC	−0.869^*∗*^	NC	NC	−0.666	NC	NC

*∗∗* means significant difference at *p* < 0.01.

*∗* means significant difference at *p* < 0.05.

NC means not computed.

**Table 4:** Antiproliferative activities of the free and bound phenolic extracts from six sesame seeds varieties against HepG2 cell.

Varieties	Antiproliferative capacity to HepG2, EC_50_ (mg mL^−1^)	
	Free phenolic extract	Bound phenolic extract
B1	66.08 ± 0.35^b^	55.49 ± 9.01^b^
B2	21.04 ± 0.60^a^	23.57 ± 0.88^a^
B3	63.45 ± 0.85^b^	30.06 ± 0.82^a^
W1	87.29 ± 3.57^c^	77.74 ± 1.29^c^
W2	82.12 ± 3.10^c^	109.53 ± 1.23^d^
W3	124.91 ± 2.79^d^	67.18 ± 1.92^c^

Data were reported as mean ± SD.

Values in the same rows with different letters differ significantly at *p* < 0.05.
