# Supplementary material for: Phytochemical Contents and Antioxidant and Antiproliferative Activities of Selected Black and White Sesame Seeds
Source: Biomed Res Int. 2016 Aug 14;2016:8495630. doi: 10.1155/2016/8495630 (PMC5002301; doi:10.1155/2016/8495630)
Supplement: Supplementary file 1 — The inhibition of HepG2 cells proliferation by the free and bound fractions were observed in a dose-dependent manner in Figure S1. The EC50 of free phenolic extracts ranged from 21.04 ± 0.60 to 124.91 ± 2.79 mg mL− 1 for HepG2 cells, while corresponding values for bound phenolic extracts ranged from 23.57 ± 0.88 to 109.53 ± 1.23 mg mL− 1. Lower values of EC50 indicate a higher antiproliferative activity. Both free and bound phenolic extracts of B2 variety showed highest antiproliferative activity. [file 8495630.f1.pdf]

Supplementary file

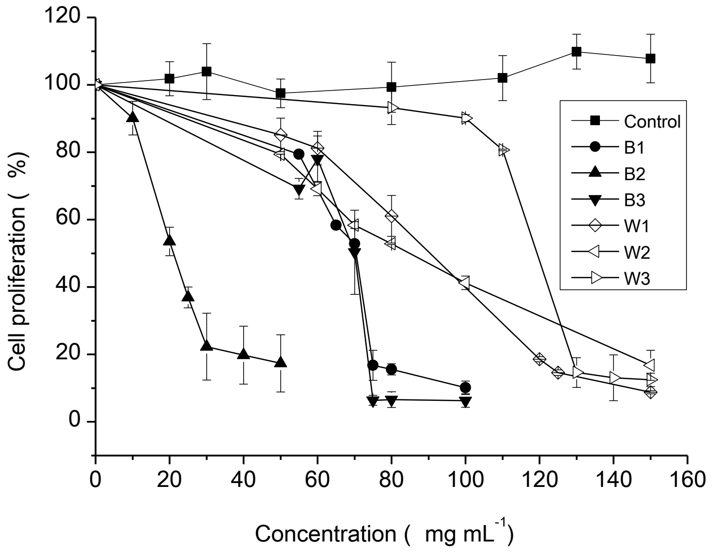

(a)

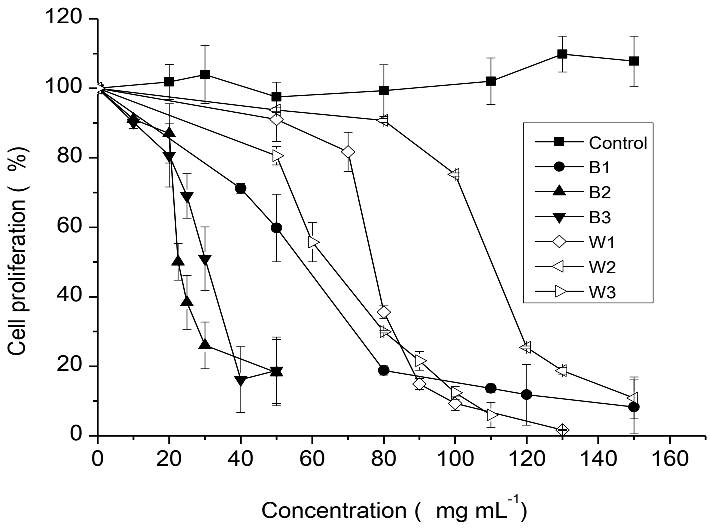

(b)

Figure S1. Antiproliferative activities of free and bound fractions of six sesame seeds varieties (B1, B2, B3, W1, W2, W3) against HepG2 human liver cancer cells (means ± SD, n = 3). (a). free phenolic extract; (b) bound phenolic extract.
